# Carcinosarcomas of the larynx: systematic review of the literature of a rare nosologic entity

**DOI:** 10.1007/s00405-021-07027-6

**Published:** 2021-08-05

**Authors:** Andrea Colizza, Antonio Gilardi, Antonio Greco, Fabrizio Cialente, Federica Zoccali, Massimo Ralli, Antonio Minni, Marco de Vincentiis

**Affiliations:** 1 Department of Sense Organ, Sapienza University of Rome, Viale del Policlinico 155, 00186 Rome, Italy; 2 Department of Oral and Maxillofacial Sciences, Sapienza University, Rome, Italy

**Keywords:** Spindle cell carcinoma, Laryngeal carcinosarcoma, Head and neck malignancies

## Abstract

**Purpose:**

Carcinosarcoma, also known as Spindle Cell Carcinoma (SpCC), is a rare type of malignant tumor. Generally, this type of pathology occurs in the urogenital tract, the gastrointestinal tract, respiratory tract and mammary gland; in the larynx, SpCC represents only 2–3% of all malignancies. Due to its rarity, there is currently no generally acceptable treatment guideline for this disease. The aim of this study was to systematically review the literature of SpCC of larynx and report epidemiologic, clinicopathologic and main therapeutic approaches for this entity.

**Methods:**

A systematic literature review was performed using MEDLINE, EMBASE, PubMed and Scopus databases. For this review, the results were extrapolated in the period between January 1990 to September 2020. Data extraction was performed using a standard registry database. The clinical and pathological staging were recalculated according to the Eight Edition of AJCC Cancer Staging Manual and statistical analyses were performed using SPSS Version 25.0.

**Results:**

A total of 111 patients affected by laryngeal carcinosarcoma were included. From our review arises that surgery is the main treatment for primary laryngeal carcinosarcoma. In this way, various techniques such as minimally invasive laryngoscopy excision, laser CO2 cordectomy, partial laryngectomy (vertical and horizontal) and total laryngectomy. The role of radiotherapy is still controversial. The overall survival (OS) for T1 stage tumor at 5 years of follow-up is 82.9%, the OS for T2 and T3 tumor is 74% and 73.4%. The OS at 5 years of follow-up is 91.7% for supraglottic tumor, 69.3% for glottic tumor and 50% for transglottic site. Subglottic site is described in only 2 cases [12–13], so the OS at 5 years is not statistically significant. The 5-year overall survival in patients without lymph nodes involvement (N0) is 90.2%, 66.7% and 50%, respectively, for N1 and N2 lesions.

**Conclusion:**

Primary laryngeal carcinosarcoma is a very rare malignancy. There are no clear guidelines in the management but in the literature, surgery is described as the best modality of therapy; radiation only can be a reasonable alternative with controversial efficacy. The most important prognostic factor is the nodal metastasis.

## Introduction

Carcinosarcoma, also known as Spindle Cell Carcinoma (SpCC), is a rare type of malignant tumor. It is an unusual form which consists of elongated (spindle) epithelial (carcinomatous) and mesenchymal (sarcomatous) components, which made the tumor biphasic [1, 2]. This is an uncommon phenomenon arising from an unclear etiology. For these reasons, different terms have been used to describe it, such as carcinosarcoma, pseudosarcoma, sarcomatoid carcinoma, pleomorphic carcinoma and spindle cell carcinoma [3]. Generally, this type of pathology occurs in the urogenital tract, the gastrointestinal tract, respiratory tract and mammary gland [4]. The most involved sites of head and neck area are the parotid and the submandibular gland [5]. In the larynx, SpCC represents only 2–3% of all malignancies [6]; the most common site is the glottic region followed by supraglottic region. Laryngeal SpCCs are predominantly in middle-aged to elderly men and have a strong association with tobacco and alcohol use [3]. The most frequent symptoms at the presentation are dysphonia, hoarseness and rarely dyspnea, when arising in subglottic site. Macroscopically, the majority of cases appear as exophytic and polypoid mass arising from one of the true vocal folds or from anterior commissure [7]. Due to its rarity, there is currently no generally acceptable treatment guideline for this disease. The aim of this study was to systematically review the literature of SpCC of larynx and report epidemiologic, clinicopathologic and main therapeutic approaches for this rare entity.

## Materials and methods

### Literature search strategy

Following the Preferred Reporting Items for Systematic and Meta-Analyses (PRISMA) guidelines, a systematic literature review was performed using MEDLINE, EMBASE, PubMed and Scopus databases. The search strategy was conducted using combinations of the following terms: carcinosarcoma OR spindle cell carcinoma OR sarcomatoid carcinoma OR collision tumor OR pseudosarcoma AND larynx.

### Study selection

For this review, the results were extrapolated in the period between January 1990 and September 2020. All the clinical series and case reports included a reported abstract available in English language, cancer site and staging, treatment options, oncological outcome and follow-up on individual patients. We excluded articles with lacking information regarding stage of tumor, therapy, outcome and time of follow-up.

Title and abstract were watchfully examined by two authors (A.C and A.G) independently, and disagreements were resolved by a discussion with a third author (M.R).

### Data extraction

The full text of the included studies was reviewed and data extraction was performed using a standard registry database. Epidemiologic and clinicopathologic data, registered in each case, included age, sex, clinical features, location, stage, type of surgical intervention, neoadjuvant or adjuvant treatment, recurrence, metastasis and survival. The clinical and pathological staging were recalculated according to the Eight Edition of AJCC Cancer Staging Manual [8]. All statistical analyses were performed using SPSS Version 25.0 (IBM Corp, Armonk, NY).

### Study quality

The levels of evidence according to the standards by Wasserman et al. [9] of the included articles were scored as follows: level I: randomized controlled trials; level II: prospective study with internal control group; level III: retrospective study with internal control group; level IV: case series without an internal control group; and level V: consensus or expert opinion without critical appraisal.

## Results

### Search results

**Fig. 1:**
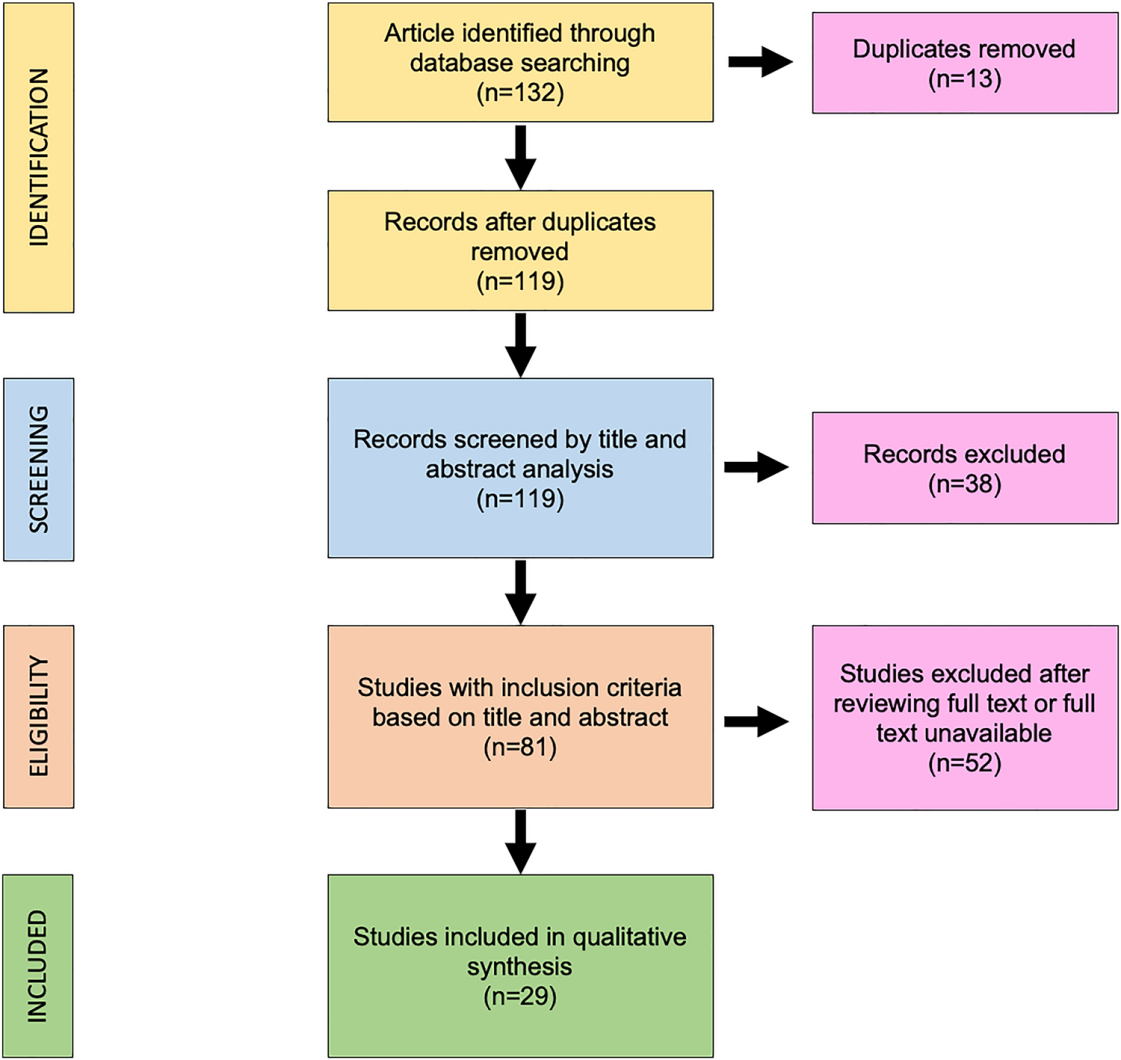
Preferred Reporting Items for Systematic Reviews and Meta-Analyses (PRISMA) diagram followed in this review. The diagram shows the information flow through the different phases of the review and illustrates the number of records that were identified and included

The search algorithm and review results are outlined in Fig. 1.

The initial search found 132 studies on the MEDLINE database, EMBASE, Scopus and the Cochrane Library databases. The removal of duplicates identified 119 publications. All the 119 papers were screened in title and abstract, and 81 manuscripts were reviewed in full text. Of these, 29 studies met the inclusion criteria, while the remaining 52 studies were excluded. The included studies were published in peer-reviewed journals without an internal control group with level IV of evidence and retrospective study with an internal control group with level III of evidence. No randomized controlled trials were identified.

### Data synthesis and analysis

The included studies are heterogeneous, so a formal meta-analysis could not be appropriately performed. The data collected from each study were transcribed in a tabular form. A total of 111 patients affected by laryngeal carcinosarcoma were included. In Table 1 are summarized the larynx site of origin, the TNM and the AJCC stage for each case. In all the cases, TNM and staging of disease were recalculated according to the Eight Edition of AJCC Cancer Staging Manual [8].

**Table 1:** Laryngeal site of origin and TNM stage of each patients. The stage of disease is according to AJCC Eight Edition

Site tumor	
Glottis	85
Supraglottis	19
Subglottis	4
Transglottis	3
T stage	
T1	53
T2	33
T3	24
T4	1
N stage	
N0	103
N1	3
N2	4
N3	1
M stage	
M0	111
M1	0
AJCC stage	
Stage 1	53
Stage 1	31
Stage 3	21
Stage 4	6

The strategy therapy approach is resumed in Table 2 and in Table 3, there is the status of each patient at the end of follow-up.

**Table 2:** Main therapeutic approach described in studies considered in this review

Surgery on T	
Laser cordectomy	10
Endoscopic excision	18
OPHL	12
Vertical partial laryngectomy	10
Total laryngectomy	29
No surgery	30
Surgery on N	
Monolateral neck dissection	6
Bilateral neck dissection	22
No neck dissection	81
Adjuvant therapy	
Adjuvant CT	0
Adjuvant RT	17
Adjuvant CT + RT	0
No adjuvant therapy	94
Neoadjuvant therapy	
Neoadjuvant RT	1
Neoadjuvant CT	0
Neoadjuvant CT + RT	0
No neoadjuvant therapy	110
Other therapy	
Exclusive RT	30
Exclusive CT	0
Exclusive CT + RT	0

**Table 3:** Follow-up of each patient included in this review

No evidence of disease (NED)	87 (78.3%)
Died for other cause (DOC)	7 (6.3%)
Died of disease (DOD)	17 (15.4%)

## Discussion

In the literature, most publications on carcinosarcoma of the larynx are case reports and just some case series. Previous publication reported that the majority of patients with laryngeal carcinosarcoma are male (93%), the ratio of men to women is 11:2 and the median age at presentation varies from 58 to 65.6 years [10, 11]. In this review, we confirmed this data: the male represents 91% of all cases and the M:F ratio is approximately 10:1. Furthermore, we noticed that there is a wide age range for laryngeal carcinosarcoma (24–81 years) and the median age of presentation is 62.1 years, similarly the most common squamous laryngeal cancer [12]. The major risk factors described for laryngeal carcinosarcoma are the alcohol addiction, smoking, and radiation exposure [13, 14]. In this review, we found 69 smoking patients, 3 non-smoking patients and for 39 patients this information is not reported. Regarding the alcoholic use, we found 10 alcoholic patients, 9 non-alcoholic patients and in 91 patients this information is not reported.

In previous work, it was reported that approximately from 50 to 90% of these patients had a history of smoking and drinking [10].

Due to the rarity of the disease, in the literature, there are a small number of cases described in the head and neck with a mix of primary sites and stages (oral cavity, larynx, and paranasal sinuses). For these reasons, there is not a clear consensus in the management and the treatment paradigm, as for conventional squamous cell carcinomas, is surgery, radiotherapy (RT) or a combination of both [3]. From our review arises that surgery is the main treatment for primary laryngeal carcinosarcoma. In this way, various techniques such as minimally invasive laryngoscopy excision, laser CO2 cordectomy, partial laryngectomy (vertical and horizontal) and total laryngectomy. The role of radiotherapy is still controversial. In the study by Zhang et al. [5], radiotherapy is not recommended for the exclusive treatment of this type of tumor. In contrast, another study by Ballo et al. [15] suggested that radiation therapy can be used as a single treatment and can effectively treat carcinosarcoma. Typically, mesenchymal tumor cells are described as not sensitive to radiation and surgical treatment is preferred due to previously reported higher rates of local recurrence with RT [16, 17]. In this review, the chemotherapy treatment has never been administrated to any patient either as a single treatment modality or in combination with surgery or radiotherapy.

A previous study published by Thompson et al. [11] proposed that the negative factors associated with poor prognosis of laryngeal carcinosarcoma patients include tumor T stage, tumor location, vocal cord movement and history of head and neck radiation therapy. According to this work, we analyzed the influence of some factors on the prognosis, in particular the extension of primary tumor (T stage), lymph nodes metastasis (N stage) and the location of tumor.

**Fig. 2:**
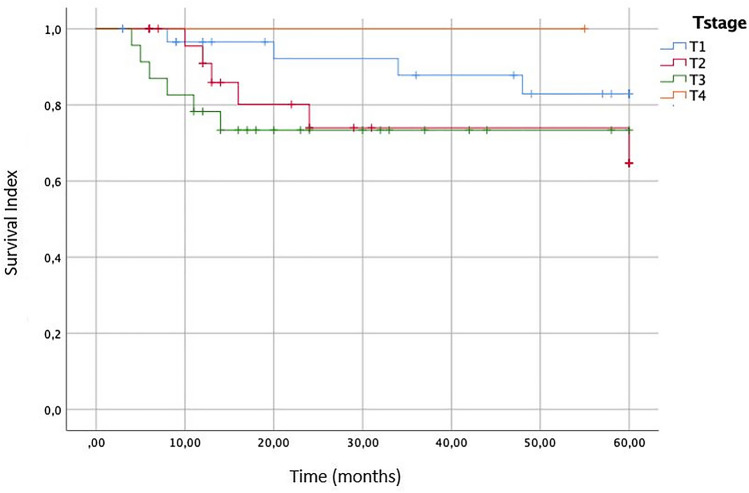
Overall survival (OS) of the patients affected by laryngeal Spindle Cell Carcinoma according to different T stages of the lesion

About the influence of T stage, we performed a Kaplan–Meier survival curve (Fig. 2). The overall survival (OS) for T1 stage tumor at 5 years of follow-up is 82.9%, the OS for T2 and T3 tumor is 74% and 734%. Probably the similar prognosis for this T2 and T3 stage is secondary to an aggressive surgical approach for T3 cancer.

The overall survival outcomes of similarly staged conventional squamous cell carcinoma of the larynx at 5 years for stage I is 84%, versus 82.9% for the same stage of SpCC emerged from this review. For other stages (II–IV), the 5-year overall survival rates were 74% for sarcomatoid tumors versus 51% for squamous cell carcinomas [3, 18].

**Fig. 3:**
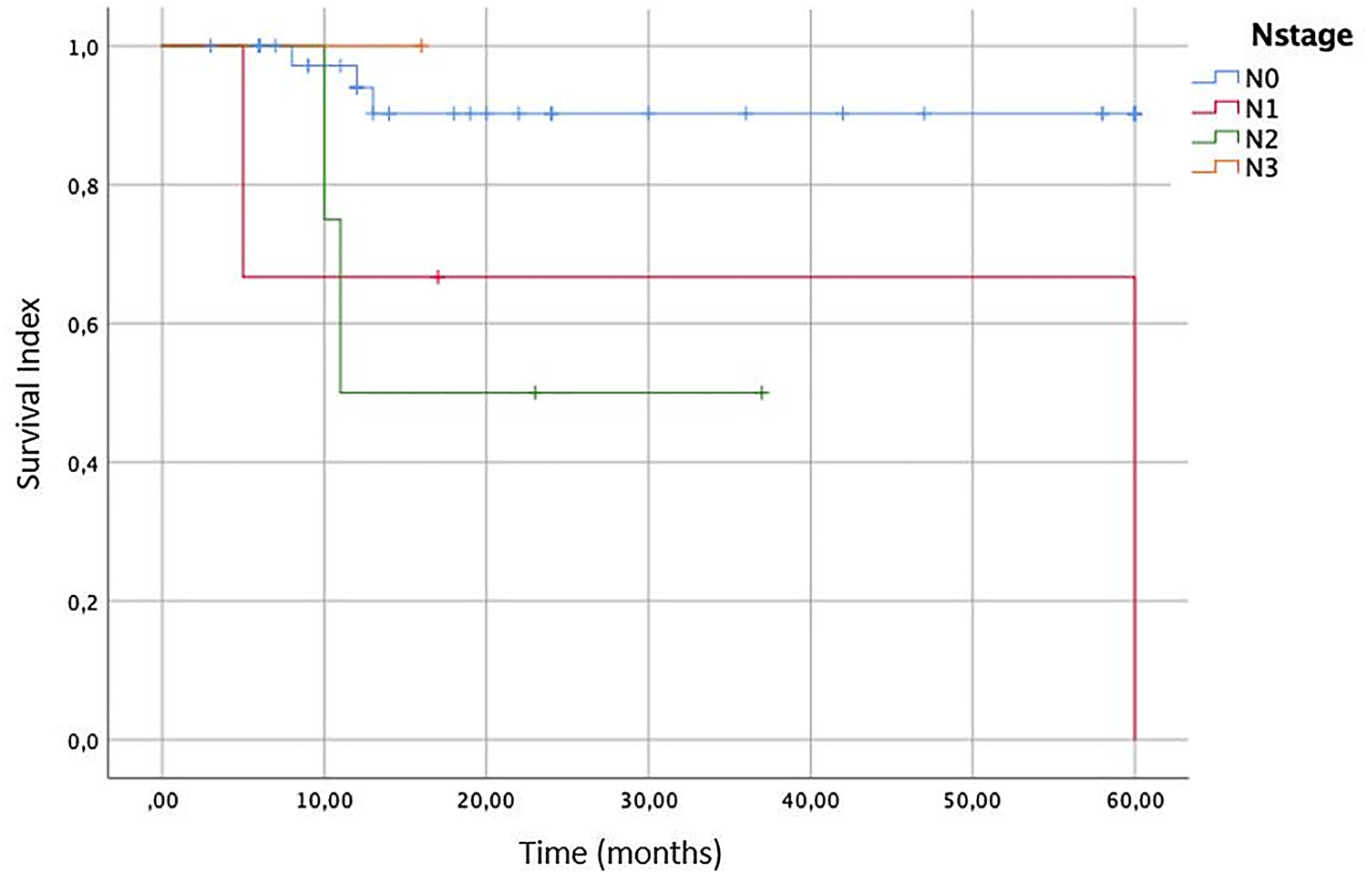
Overall survival (OS) of the patients affected by laryngeal Spindle Cell Carcinoma according to different N stages of lymph nodes metastasis

In the literature, the lymph node metastasis were not very common for SpCC but, according to some authors, there is the tendency to metastasize to the opposite side of the neck. In fact, in our review, we found the nodal involvement in 8/111 total patients. In Fig. 3 is represented a Kaplan–Meier survival curve according to the presence of metastasis in cervical lymph nodes. We found that the 5-year overall survival in patients without lymph nodes involvement (N0) is 90.2%, 66.7% and 50%, respectively, for N1 and N2 lesions. The N3 stage is reported in only one case [19], so the OS survival is not statistically significant. According to this survival analysis, it is clear the importance of regional metastasis.

**Fig. 4:**
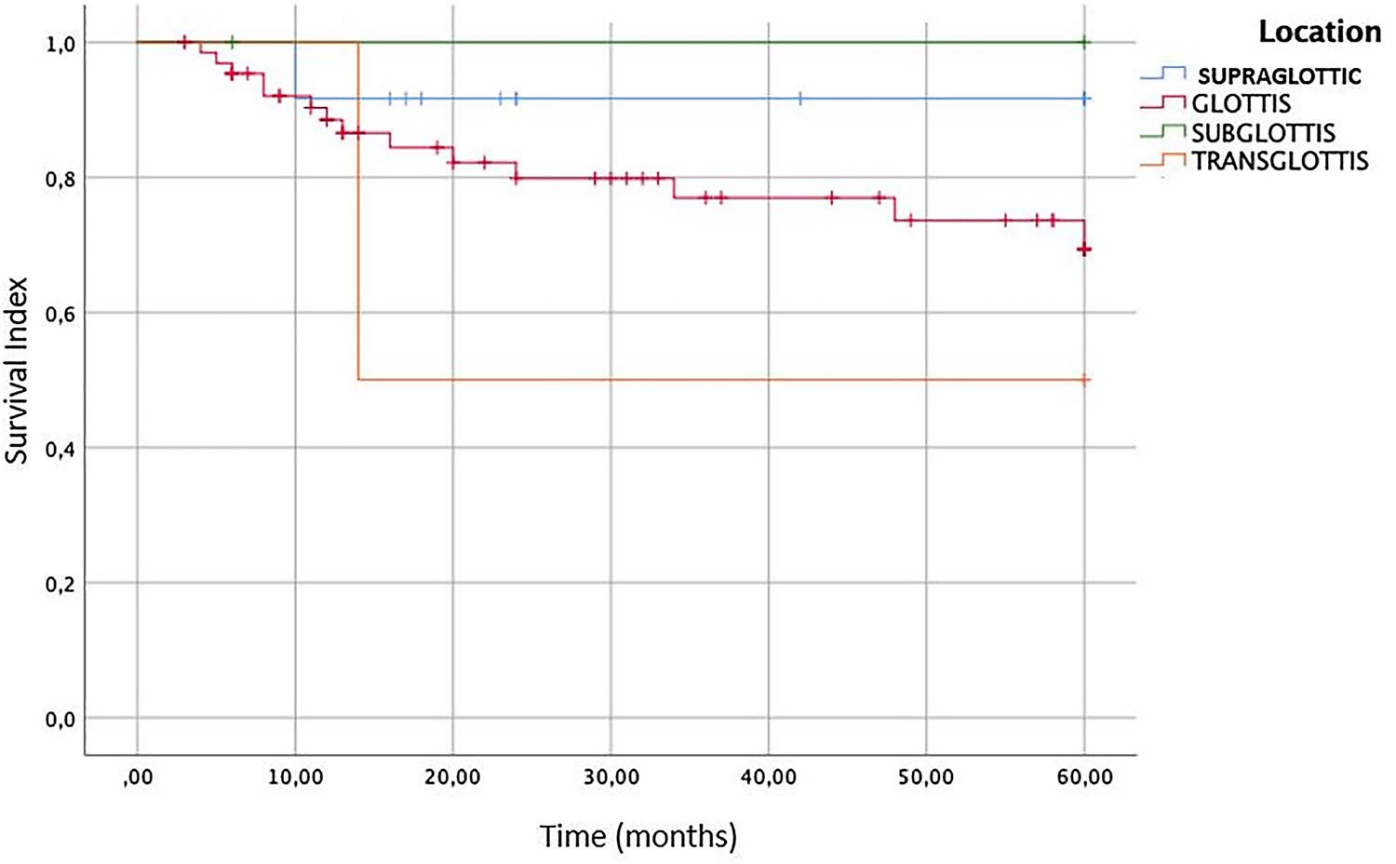
Overall survival (OS) of the patients affected by laryngeal Spindle Cell Carcinoma stratified to different sites of origin of the cancer

The location site of the tumor is another important prognostic factor described in the literature. The Kaplan–Meier survival curve is reported in Fig. 4. The OS at 5 years of follow-up is 91.7% for supraglottic tumor and 69.3% for glottic tumor. The subglottic site is described in only 2 cases [13–20], so the OS at 5 years is not statistically significant. Very interesting is the OS for the transglottic tumor; for this site, the OS is 50%, extremely worse than the other subsite of larynx.

## Conclusion

Primary laryngeal carcinosarcoma is a very rare malignancy. It can present at an early age and diagnosis is possible in the early stages. There are no clear guidelines in the management but in the literature, surgery is described as the best modality of therapy; radiation only can be a reasonable alternative with controversial efficacy. The most important prognostic factor is the nodal metastasis. The main characteristics of this type of tumor are the tendency to metastasize to the other side of the neck.
